# The Cytoskeletal Protein Ndel1 Regulates Dynamin 2 GTPase Activity

**DOI:** 10.1371/journal.pone.0014583

**Published:** 2011-01-25

**Authors:** Mathieu Chansard, Jian Wang, Hong Chi Tran, Gernot Neumayer, Su Yeon Shim, Young-Un Park, Camille Belzil, Hoa Thi Le, Sang Ki Park, Minh Dang Nguyen

**Affiliations:** 1 Departments of Clinical Neurosciences, Cell Biology & Anatomy, and Biochemistry & Molecular Biology, Hotchkiss Brain Institute, University of Calgary, Calgary, Alberta, Canada; 2 Division of Molecular and Life Science, Department of Life Science, Pohang University of Science and Technology, Pohang, Republic of Korea; 3 Division of Integrative Biosciences and Biotechnology, Pohang University of Science and Technology, Pohang, Republic of Korea; Iowa State University, United States of America

## Abstract

Cytoskeleton dynamics, membranes trafficking and positioning are essential for the proper functioning of any mammalian cell. The identification of the molecules and mechanisms that allow these cellular processes to interface is vital for understanding cell behaviors. Ndel1, the mammalian homolog of the *Aspergillus nidulans* NudE, organizes the cytoskeleton and regulates molecular motors, thereby impacting on the positioning of membranes. Hypothetically, Ndel1 can act in concert with enzymes controlling membrane trafficking (vesicle-mediated transport) per se, but this idea has never been investigated. We now report that a pool of Ndel1 associates directly with Dynamin 2 (Dyn2), a large cytosolic GTPase involved in the trafficking of the AMPA receptor subunit GluR1. In vitro, Ndel1 enhances Dyn2 GTPase activity in its unassembled and assembled forms, without promoting oligomerization of the enzyme. In cells, gain and loss of function of Ndel1 recapitulate the effects of overexpression of Dyn2 and Dyn2 dominant negative with reduced GTPase activity on the intracellular localization of GluR1, respectively, without affecting the stability of microtubules. Together, these results indicate that Ndel1 regulates Dyn2 GTPase activity and impacts GluR1-containing membranes distribution in a manner reminiscent of Dyn2.

## Introduction

Composed of microfilaments (MFs), Intermediate Filaments (IFs), Microtubules (MTs) and their associated proteins, the cytoskeleton is the internal scaffolding that provides structure for the cell, as well as transports materials and sends signals across the cell. Emerging evidence indicate that Ndel1, a 345 amino-acid coiled-coil protein and the mammalian homolog of the *Aspergillus nidulans* NudE, organizes the cytoskeleton and regulates molecular motors in numerous cell types [1]–[7]. In mitotic cells, through association with MTs, Ndel1 ensures the assembly of the mitotic spindle, centrosomal maturation and mitosis [8], [9]. During mitosis, Ndel1 also regulates the alignment and segregation of chromosomes. In the developing neocortex, Ndel1, in association with the Dynein motor and the lissencephaly protein Lis1, contributes to neuronal migration [8], [10]–[12] (for a review on neuronal migration, refer to [13]). It does so by stabilizing MTs and promoting nucleokinesis, the process that pulls the nucleus toward the extending leading process of a migrating neuron [12]. In addition, Ndel1 can also influence actin organization and dynamics through interactions with Rho GTPases and Paxillin during cell migration and adhesion [14]–[17]. Finally, Ndel1 induces neuronal differentiation and maintains cell integrity of maturing neurons through polymerization of neuronal IFs (neurofilaments) transported by Dynein and Kinesin [1], [18], [19]. In each of these biological processes, Ndel1 not only plays a key role in maintaining structural integrity but also appears to position organelles and traffic membranes via MTs and molecular motors. For instance, Ndel1 participates in the positioning of Golgi membranes through the MTs/Lis1/Dynein pathway [20], [21]. Whether Ndel1 contributes to membrane trafficking (vesicle-mediated transport) per se through other mechanisms such as by regulating proteins that shape membranes structure, remains however undefined.

Dynamin (Dyn) is a large cytosolic GTPase (∼100 kDa) that was first isolated from the brain as a microtubule-binding protein, although little evidence points to a role for Dyn in MTs remodelling [22]. However, a recent study indicates that Dyn2 is involved in dynamic instability of MTs [23]. Dyns are most well characterized for their action on membranes. Dyn associates with membranes and through oligomerization into ring-like structures, wraps around the neck of budding vesicles [22], [24], [25]. Following hydrolysis of GTP, Dyn changes its conformation to constrict and pinch membranes [22], [24]. A longitudinal tension appears to be required to pull apart the membranes and allow membrane fission during membrane trafficking in yeast, vertebrate and mammalian cells [25]–[27].

There are three isoforms of Dynamin: Dyn1, Dyn2 and Dyn3 [22], [28]. Whereas Dyn1 and Dyn3 are expressed in a tissue-specific manner, Dyn2 is ubiquitously expressed [29]. Dyn2 is enriched in clathrin-coated pits at the plasma membrane [30]. A very minor fraction of Dyn2 is presumably found at the trans-Golgi network (TGN) [31], [32]. Dyns have also been linked to actin dynamics and implicated in calveolae internalization, vesicles recycling at the synapse, lamellipodia formation, cell migration and invasion [33]–[41]. In a yeast two-hybrid screen, we recovered Dyn2 as a Ndel1-binding partner. We verified this interaction biochemically and molecularly. We also found that Ndel1, like Dyn2, impacts the intracellular distribution of the á-amino-3-hydroxyl-5-methyl-4-isoxazole-propionate (AMPA) receptor GluR1, possibly through regulation of Dyn2 GTPase activity.

## Results

### Ndel1 interacts directly with Dynamin 2

**Figure 1:**
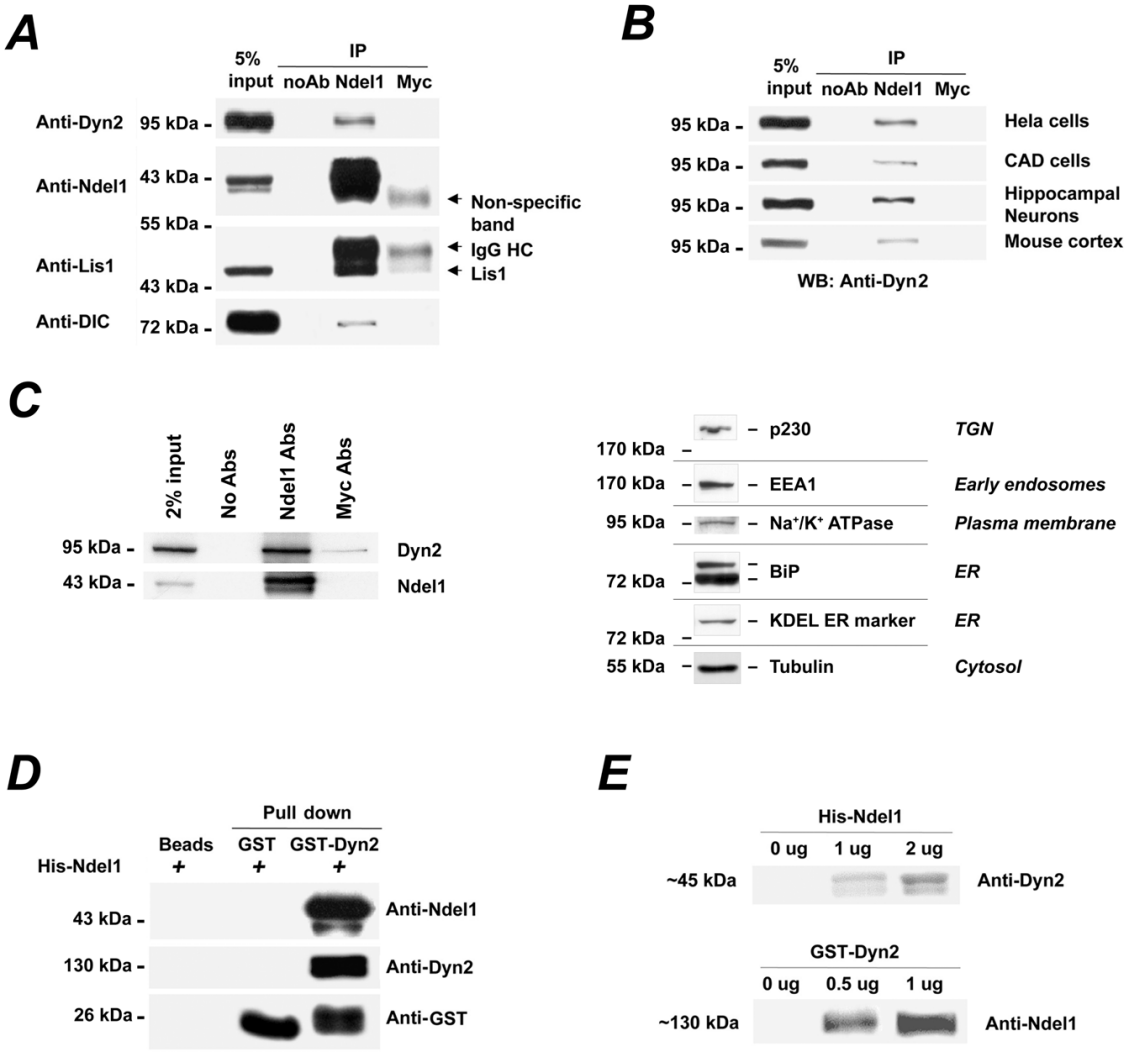
Interaction between Ndel1 and Dyn2. (A) Dynamin2 (Dyn2) co-immunoprecipitates with Ndel1, so do Lis1 and Dynein intermediate chain (DIC) in HeLa cells. Co-immunoprecipitations in absence of antibody (noAb) or with Myc antibody serve as negative controls. (B) Dyn2 co-immunoprecipitates with Ndel1 in diverse cell types (HeLa cells, neuroblastoma CAD cells, primary cultured rat hippocampal neurons) and mouse cortex. (C) Co-immunoprecipitation of Dyn2 with Ndel1 in a post-mitochondrial fraction from HeLa cells (left panel). Co-immunoprecipitations in absence of antibody (No Abs) or with Myc antibody serve as negative controls. The right panel presents a Western blot analysis of the membranes content of a post-mitochondrial fraction. Endoplasmic reticulum (ER), trans-Golgi (TGN), plasma membrane, endosomes and cytosolic proteins are all present in this fraction, as detected by KDEL ER marker/BiP, p230 trans-Golgi, Na^+^/K^+^ ATPase, EEA1 and Tubulin antibodies, respectively. (D) In vitro pull-down of His-Ndel1 by GST-Dyn2 but not GST. The protein detected by anti-GST antibodies in GST-Dyn2 pull-down corresponds to the cleaved GST from GST-Dyn2. (E) Far-western assays demonstrating the direct interaction between Ndel1 and Dyn2. His-Ndel1 bound to membranes was overlaid with GST-Dyn2 protein that was detected with a Dyn2 antibody at the His-Ndel1 molecular weight (∼45 kDa). Consistently, GST-Dyn2 bound membranes overlaid with His-Ndel1 that was detected with a Ndel1 antibody at the GST-Dyn2 molecular weight (∼125 kDa = ∼100 kDa for Dyn2+∼25 kDa for GST).

In a yeast two-hybrid screen, we recovered Dyn2 as a Ndel1-binding partner. To determine whether Dyn2 forms a complex with the MT-associated factor Ndel1 in cells, co-immunoprecipitation experiments with Ndel1 antibodies were performed on HeLa cell lysates. As shown in Fig. 1A, Dyn2 co-immunoprecipitated with Ndel1 as did Lis1 and Dynein. The association between Ndel1 and Dyn2 was also detected in neuroblastoma CAD cells, rat primary cultured hippocampal neurons and adult mouse cortex (Fig. 1B). All co-immunoprecipitation experiments were controlled with the absence of antibodies or the presence of Myc antibodies. Using a post-mitochondrial fraction (PMF), an intermediate fraction composed of cytosol, Golgi, endoplasmic reticulum (ER), endosomes and plasma membranes, we succeeded in co-immunoprecipitating Dyn2 with Ndel1 (Fig. 1C). Taken together, these results indicate that Dyn2 forms a complex with Ndel1 in vivo.

The direct interaction between Ndel1 and Dyn2 was demonstrated by an in vitro binding assay using purified His-Ndel1 and GST-Dyn2 fusion proteins. While GST protein did not associate with His-Ndel1, GST-Dyn2 pulled down His-Ndel1 (Fig. 1D). The direct interaction between the two proteins was further tested by Far-western assays. Incubation of GST-Dyn2 with membrane-bound His-Ndel1 revealed specific binding of Dyn2 to Ndel1 at the expected molecular weight (∼45 kDa). The binding occurred in a dose-dependent manner, as increasing the amounts of His-Ndel1 (1 to 2 µg) resulted in stronger signals with Dyn2 antibodies (Fig. 1E). Lanes with no protein loaded did not exhibit immunoreactivity. In the reverse experiment, incubation of soluble His-Ndel1 protein with membrane-bound GST-Dyn2 verified the direct interaction between the two proteins (Fig. 1E).

**Figure 2:**
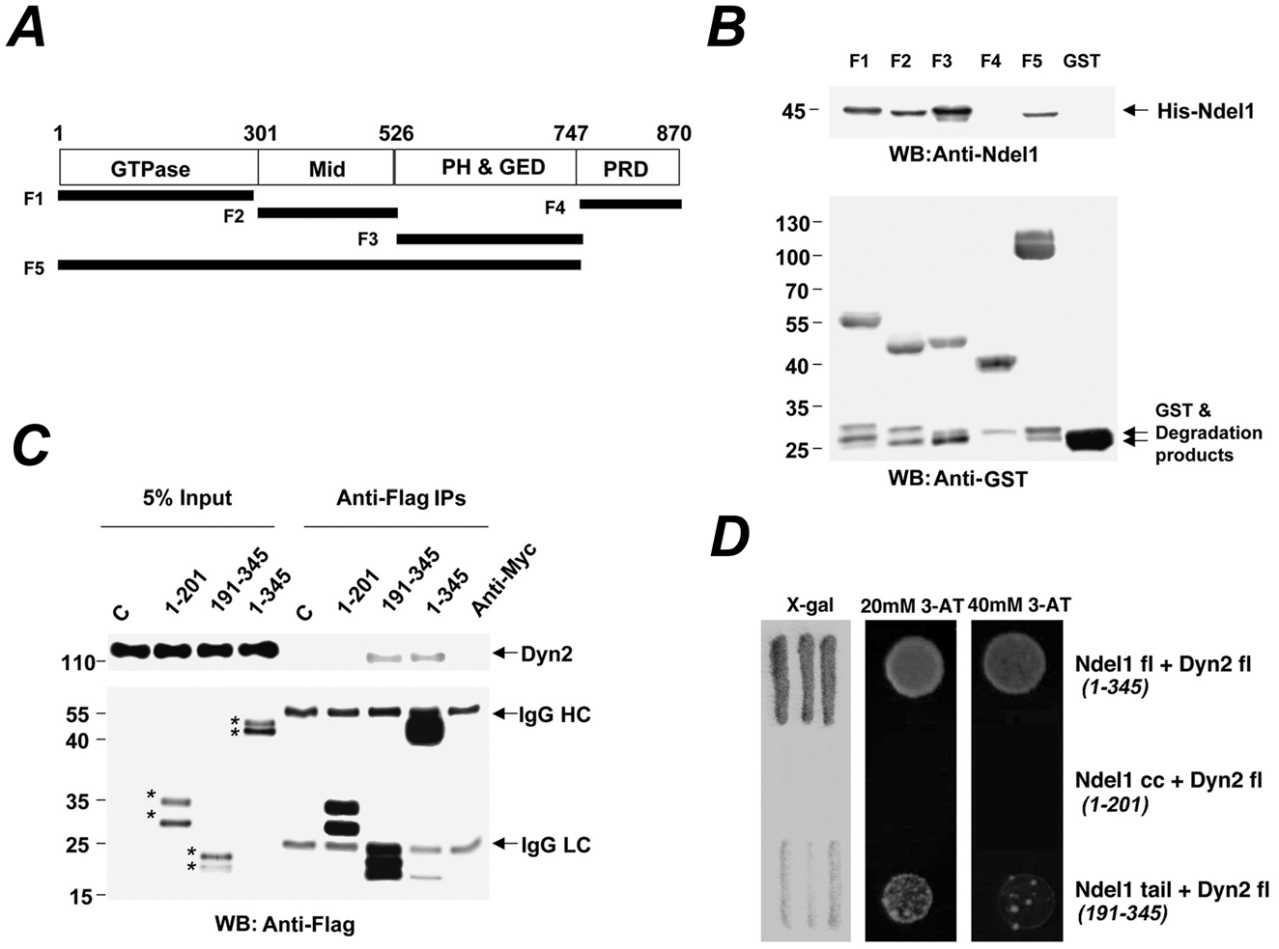
Ndel1 interacts with Dyn2 on multiple domains. (A) GST-tagged constructs of Dyn2 used for GST pull-downs. Mid, middle domain; PH, pleckstrin homology domain; GED, GTPase effector domain; PRD, proline-rich domain. (B) GST pull-downs experiments indicating that all domains in Dyn2 with the exception of the PRD bind to His-Ndel1. (C) Endogenous Dyn2 co-immunoprecipitates with Ndel1 full length (fl) (a.a. 1–345) and C-terminus (tail) (a.a. 191–345) in transfected HeLa cells. *Bands that have been confirmed with Ndel1 antibodies. C, cells transfected with an empty vector. (D) Dyn2 full length interacts with Ndel1 full length (a.a. 1–345) and Ndel1 tail (a.a. 191–345) but not Ndel1 coiled-coil domain (a.a. 1–201) in yeast 2-hybrid assays as detected by X-Gal and 3-AT.

Like other Dynamins, Dyn2 is composed of a GTPase domain, a middle domain, critical for tetramerization and high-order self-assembly, a Pleckstrin Homology domain (PH) for membrane association, a GTPase Effector Domain (GED) that acts as an intramolecular GTPase Activating Protein (GAP) and a Proline-Rich Domain (PRD) that varies among members of the Dyn family and indirectly promotes cell signalling through association with various protein partners bearing SH3 domains [22]. To map the domain(s) of interaction between Ndel1 and Dyn2, recombinant His-Ndel1 and truncated GST fragments of Dyn2 were expressed and purified for in vitro binding assays (Fig. 2A). The GTPase domain, the middle domain, the PH and GED domains, but not the PRD of Dyn2 interacted with His-Ndel1 (Fig. 2B). The F5 construct containing all three binding sites bound less to Ndel1 than the individual F1-F3 constructs suggesting possible conformational changes in Dyn2 and/or competition between multiple binding sites. To map the interaction domain(s) on Ndel1, N-terminus (a.a. 1–201), C-terminus (a.a. 191–345) or full length (a.a. 1–345) Flag-Ndel1 constructs were transfected in HeLa cells and co-immunoprecipitation experiments were performed with Flag antibody. The C-terminus and full length Ndel1, but not the N-terminus, interacted with endogenous Dyn2 (Fig. 2C). This Ndel1 tail/Dyn2 direct interaction was confirmed by yeast two-hybrid assays (Fig. 2D). Together, these results indicate that the C-terminus of Ndel1 is sufficient for direct interaction with Dyn2, possibly with all domains excluding the PRD.

### Ndel1 enhances Dynamin 2 GTPase activity in vitro

**Figure 3:**
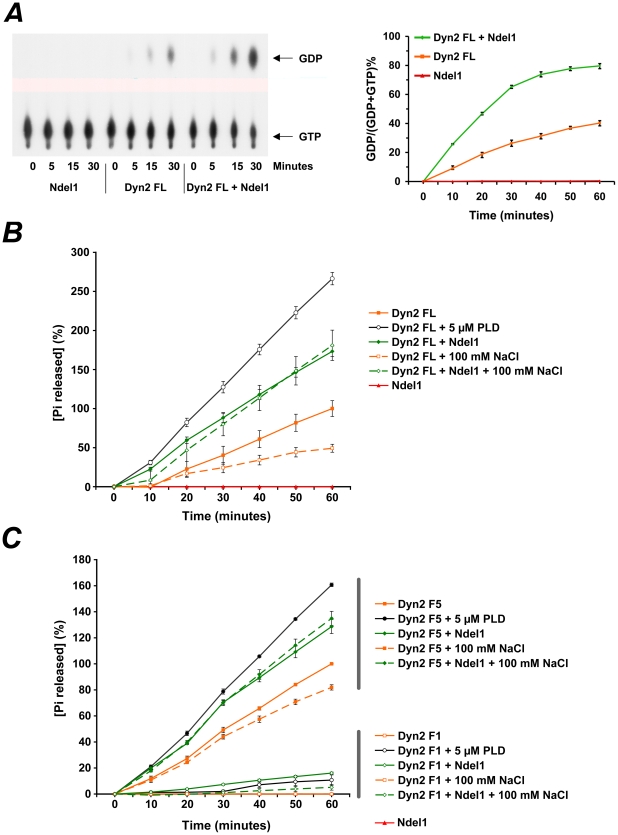
Ndel1 increases the GTPase activity of Dyn2 in vitro. (A) Radioactive GTPase assays for Ndel1 (red), Dyn2 full length (FL, orange) and Ndel1 together with Dyn2 FL (green). Ndel1 itself does not have intrinsic GTPase activity. The addition of Ndel1 to oligomerized Dyn2 FL enhances the activity of the latter by ∼2 fold over 60 minutes. Error bars indicate s.d. (n = 4). Two-way ANOVA: p<0.0001. (B) Non-radioactive GTPase assays for Dyn2 FL. Ndel1 does not show detectable GTPase activity, but increases the GTPase activity of assembled Dyn2 FL. The activator Phospholipase D (PLD, positive control) also increases the activity of oligomerized Dyn2 FL. Under high salt conditions (negative control), unassembled Dyn2 FL shows reduced GTPase activity when compared to assembled Dyn2 FL. The presence of Ndel1 enhances Dyn2 FL GTPase activity even under high salt conditions. Error bars indicate S.E.M. (n = 3). Two-way ANOVA: p<0.0001 for all conditions. (C) Non-radioactive GTPase assay for F1 and F5. Ndel1 itself does not have detectable intrinsic GTPase activity. The addition of Ndel1 increases the GTPase activity of oligomeric F5. PLD and high salt conditions elevates and diminishes F5 GTPase activity, respectively. The presence of Ndel1 enhances F5 activity even under high salt conditions. Note that the GTPase activity of F1, which cannot forms oligomers, is below detectable levels but becomes recordable following addition of Ndel1 or PLD. Error bars indicate S.E.M. (n = 3). Two-way ANOVA: p<0.0005 for all conditions.

The above findings showing that Ndel1 binds to Dyn2 through its regulatory domains including the GTPase domain (Figs. 2A, 2B) raised the question as to whether Ndel1 regulates Dyn2 GTPase activity. To test whether Ndel1 modulates Dyn2 activity, we performed two different in vitro GTPase assays. Previous studies have shown that Dyn2 activity is stimulated by oligomerization of the enzyme [22], [42], [43]. Based on these reports, we reconstituted Dyn2 oligomers in vitro under low salt conditions and measured the GTPase activity upon addition of GTP and in the presence or absence of Ndel1 (see Materials and Methods). We found that addition of Ndel1 to the reaction mixture enhanced the hydrolysis of GTP into GDP by ∼2 fold, in a time-dependent manner (Fig. 3A). In this radioactive assay, Ndel1 itself did not exhibit any GTPase activity (Fig. 3A).

Using a non-radioactive assay we further confirmed that Ndel1 augments the GTPase activity of assembled Dyn2 (Fig. 3B). Consistent with data obtained with the radioactive assay, we also confirmed that Ndel1 shows no detectable intrinsic activity (Fig. 3B). Phospholipase D (PLD), an activator of Dyn2 GTPase activity, and high salt conditions that disassemble Dyn2 oligomers, were used as positive and negative controls, respectively (Fig. 3B and [43], [44]). Interestingly, even under high salt conditions, we found that Ndel1 significantly augments Dyn2 basal activity (Fig. 3B and see below). Taken together, these results indicate that Ndel1 enhances the GTPase activity of unassembled and assembled Dyn2.

The F1 and F5 fragments of Dyn2 contain the GTPase domain, and the GTPase+middle+PH+GED domains, respectively. Both fragments have detectable activity (Fig. 3C). We further tested whether Ndel1 impacts the activity of F1 and F5. We found that Ndel1 enhances the activity of F5 by ∼1.3 fold. This increase in activity is within the same magnitude as the augmentation observed with the full length protein (Figs. 3B, 3C). Remarkably, Ndel1's effect was maintained when oligomerization of F5 was prevented under high salt conditions (Fig. 3C). In contrast to F5 and Dyn2 full length, F1, which cannot oligomerize, did not display detectable levels of activity (Fig. 3C+[44]). However, the addition of Ndel1 or PLD (positive control) elevated the GTPase activity to detectable levels. Thus, our data confirmed the existence of a low intrinsic activity of the GTPase domain (F1) that can be stimulated by PLD [44], and revealed that Ndel1 can also increase this activity. In sum, these results indicate that Ndel1 can augment the basal (unassembled) and oligomeric (assembled) GTPase activity of Dyn2 in vitro.

### Ndel1 decreases the oligomerization of Dynamin 2

**Figure 4:**
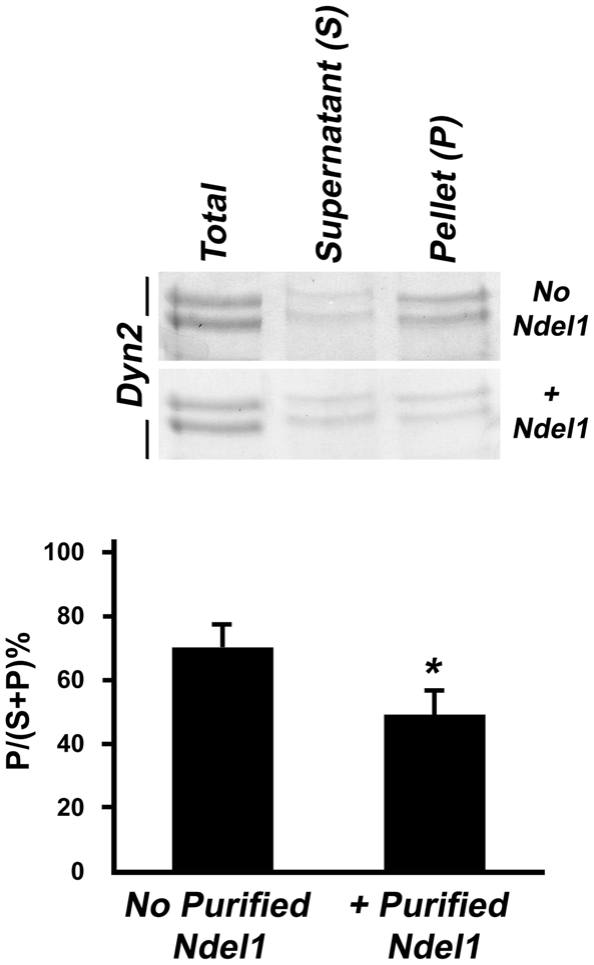
Ndel1 decreases oligomerization of Dyn2. A sedimentation assay for Dyn2 with or without Ndel1 detects lower amounts of Dyn2 in pellet of samples with Ndel1 protein vs without Ndel1 protein. Error bars indicate s.d. (n = 3). Student *t*-test: *, p<0.05.

We found that Ndel1 increases the GTPase activity of Dyn2 in its oligomeric form. We also made the observation that Ndel1 counteracts the decrease in activity caused by high salt conditions and we attributed this to an enhancement in basal activity. However, Dyn2 self-assembly per se has been proposed as a major regulator of its GTPase activity [43]. Since Ndel1 binds the middle domain of Dyn2, which is important for its oligomerization, it is possible that Ndel1 facilitates Dyn2 oligomerization. To determine whether Ndel1 facilitates Dyn2 self-assembly in vitro, Dyn2 oligomers were assembled in vitro in the presence or absence of Ndel1. The complexes were analyzed by a sedimentation assay that separates Dyn2 oligomers in the pellet (P) from unassembled Dyn2 in the supernatant (S) after centrifugation. Using this assay, ∼70% of Dyn2 sedimented (Fig. 4). Incubation of Ndel1 with non-assembled Dyn2 did not enhance the amount of Dyn2 recovered in the pellet (Fig. 4). In fact, at the opposite, a significantly lower amount of Dyn2 sedimented in presence of Ndel1. These results indicate that Ndel1 decreases Dyn2 oligomerization. They are consistent with the findings that Ndel1 enhances Dyn2 GTPase activity (GTP hydrolysis) (Fig. 3) and GTP hydrolysis stimulates Dyn2 disassembly [43]. The data also confirmed that the Ndel1-mediated increase in Dyn2 activity under high salt conditions (Fig. 3B) is not related to a facilitation of Dyn2 oligomerization but rather to an increase in basal activity.

### Ndel1 mimics the effects of Dynamin 2 on GluR1 intracellular localization

We have shown that Ndel1 interacts with Dyn2 and regulates its GTPase activity in vitro. Considering that Dyn2 regulates intracellular trafficking [31], [32] and endocytosis of the AMPA receptor subunit GluR1 [45], [46], we propose that Ndel1 may regulate GluR1 intracellular distribution in a similar way to Dyn2. To verify this hypothesis, we compared the effects of exogenous Ndel1, exogenous Dyn2, Ndel1 siRNA and mutant Dyn2 with reduced GTPase activity (Dyn2(K44A)) [45]–[47] on GluR1 distribution in HeLa cells, by membrane fractionation. Our rationale is that Ndel1's gain of function would mimic the effects of exogenous Dyn2 on GluR1 localization, while loss of function of Ndel1 would recapitulate the effects of Dyn2(K44A).

**Figure 5:**
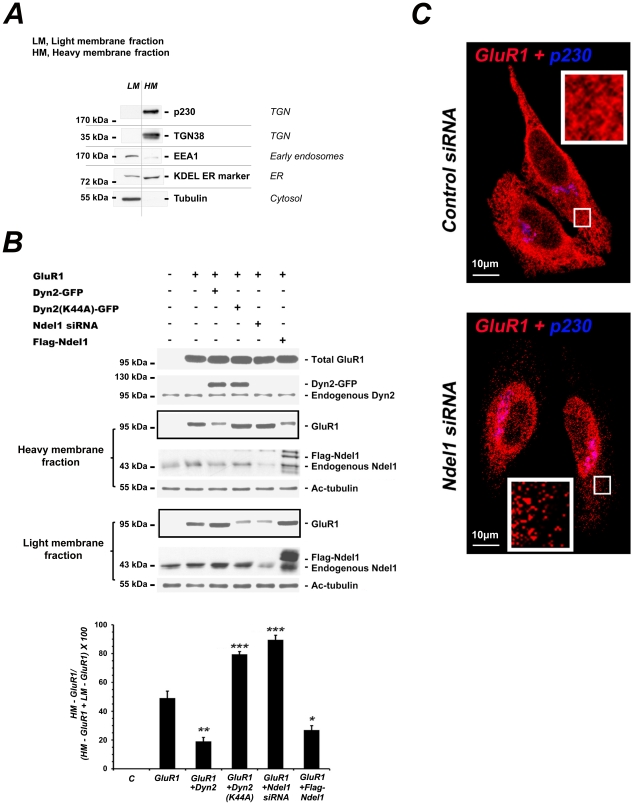
Ndel1 controls GluR1 localization in a similar way to Dyn2. Fractionation of GluR1 in heavy membranes (HM) and light membranes (LM) from HeLa cells transfected with a combination of Dyn2, GluR1, Flag-Ndel1, Dyn2(K44A) constructs and/or treated with Ndel1 siRNA. (A) The HM fraction comprises several organelles, including the trans-Golgi network (TGN) and the endoplasmic reticulum (ER), as detected with p230 trans-Golgi/TGN38 and KDEL ER marker antibodies, respectively. The LM fraction includes cytosolic proteins and small organelles like early endosomes as indicated by Tubulin and EEA1 antibodies, respectively. (B) The framed Western blots depict the separation of GluR1 in HM vs GluR1 in LM in HeLa cells. Increasing the levels of Dyn2 or Ndel1 reduces the ratio GluR1 HM/GluR1 (HM+LM), indicating GluR1 is redistributed from the heavy membranes to the lighter membranes. The usage of a dominant negative mutant of Dyn2 (Dyn2(K44A)) or the treatment of cells with Ndel1 siRNA reverts the ratio, indicating accumulating GluR1 in the HM fraction. “C” corresponds to control (cells transfected with an empty vector). Error bars indicate s.d. (n = 3). One way ANOVA: ***, p<0.001; **, p<0.01; *, p<0.05. Note that the levels of stable (acetylated) Tubulin are similar in Ndel1 siRNA-treated cells vs control siRNA-treated cells. (C) In HeLa cells treated with control siRNA, the AMPA receptor GluR1 (red) is found at the cell periphery and up to the cell edge. In HeLa cells treated with Ndel1 siRNA most of GluR1 (red) is found close to the nucleus with very little amount at the cell periphery. Cells were double stained with a marker for the trans-Golgi network (p230 trans-Golgi, blue). Scale bar, 10 µm.

Heavy membrane and light membrane fractions from the five groups of transfected cells were separated by velocity sucrose gradient (see Materials and Methods). This method of fractionation is not selective to specific organelles, as shown in Fig. 5A, but provides the general information on the localization of GluR1 in the presence or absence of functional Ndel1 or Dyn2. When expressed at low levels, GluR1 is normally found at the cell periphery (Fig. 5C). As shown in Fig. 5B, the overexpression of Ndel1 reduced the ratio of GluR1 in heavy membranes vs total levels of GluR1 (heavy membranes + light membranes = 100%) in a manner reminiscent of cells overexpressing Dyn2, when compared to cells transfected with an empty vector. These results indicate that GluR1 localization is similarly affected in the presence of an excess of Ndel1 or Dyn2, the total amount of GTPase activity being higher in these cells. Conversely, the expression of the dominant negative Dyn2(K44A) with reduced GTPase activity enhanced the ratio GluR1 (heavy membranes)/GluR1 (total levels) (Fig. 5B). Remarkably, depletion of Ndel1 by siRNA also increased the ratio GluR1 (heavy membranes)/GluR1 (total levels). The impaired localization of GluR1 in Ndel1 siRNA-treated cells is therefore similar to the distribution defects observed in Dyn2(K44A)-expressing cells. This alteration was unlikely caused by destabilization and collapse of the MT network as levels of acetylated-Tubulin (Ac-Tubulin: stable MTs) and MT structures remain essentially unchanged in Ndel1-depleted cells (Fig. 5B and references [1], [4]). Nor was it caused by remodelling or fragmentation of the TGN or endoplasmic reticulum (ER) as revealed by the fractionation profiles of the KDEL ER marker and TGN marker p230 trans-Golgi in the heavy and light membrane fractions, and their staining pattern in these Ndel1-depleted cells (Fig. S1). To further confirm the changes in GluR1 intracellular localization, we stained HeLa cells co-transfected with GluR1 at low levels and control siRNA. In these cells, GluR1 was distributed properly throughout the cell body and cell periphery (Fig. 5C). In contrast, in GluR1-expressing cells depleted of Ndel1, GluR1 mostly accumulated in perinuclear regions, with very little proteins transported to the periphery (Fig. 5C).

Taken together, these results support the notion that Ndel1 regulates GluR1 intracellular localization in a similar fashion to Dyn2. As loss of Ndel1 function mimics the effects of Dyn2(K44A) with reduced activity, these results combined with the in vitro GTPase assays suggest that Ndel1 may in part positively regulate Dyn2 GTPase activity to impact GluR1 localization.

## Discussion

We have discovered that Ndel1 is a novel regulator of the basal (unassembled) and assembled Dyn2 GTPase activity, and impacts the intracellular localization of one of Dyn2 targets, the AMPA receptor subunit GluR1. Whether Ndel1 regulates GluR1 distribution through control of Dyn2 GTPase during trafficking and/or organelles remodelling (changes in positioning/morphology) remains to be determined.

### Ndel1 is not a typical GAP

The GTPase Effector Domain (GED) of Dyns favors self-assembly and consequently, acts as an intramolecular GTPase Activating Protein (GAP) to enhance the GTPase activity of Dyn2 [48]. Several molecules such as Grb2 can indirectly stimulate the GTPase activity of Dyns by promoting self-assembly through the GED domain. In 2006, the first “external” GAP, Phospholipase D (PLD), acting directly on the active and assembled Dyns was identified [23], [42], [44]. Like other GAPs that use an arginine finger-based mechanism for activation, the PHOX homology domain of PLD contains two arginine residues essential for the GAP function. Consistent with the mechanism of other GAPs, the PHOX homology domain of PLD also interacts with the GTPase domain of Dyns in its GTP-bound state. Our biochemical experiments indicate that Ndel1 binds to the GTPase domain of Dyn2. Using in vitro radioactive and non-radioactive GTPase assays, we found that Ndel1 enhances the GTPase activity of Dyn2 in its unassembled and assembled states by a mechanism independent of the oligomerization of the enzyme. In cells, Ndel1 depletion by siRNA also mimics the effects of Dyn2(K44A), a mutant with reduced GTPase activity, while overexpression of Ndel1 impacts on GluR1 distribution in a similar way to enhancing Dyn2 activity. These results suggest that Ndel1 may regulate GluR1 intracellular distribution through Dyn2 GTPase activity. It remains unclear which of the two (unassembled or assembled) Dyn2 GTPase activities is regulated by Ndel1 in cells.

Interestingly, the interaction between Ndel1 and Dyn2 is GTP-independent (data not shown) and Ndel1 does not exhibit a PHOX homology domain. Thus, we propose that Ndel1 may act on Dyn2 in a different way than a typical GAP. Taking in consideration the relatively small molecular weight of Ndel1, its binding to all domains (GTPase, middle, PH and GED) except the PRD of Dyn2 suggests that it may adopt a particular conformation for interaction. The crystal structure of the Dyn2/Ndel1 interfaces will help define the mechanism of this novel activator.

### How does Ndel1 regulates GluR1 distribution?

Our membrane fractionation experiments indicate that Ndel1 impacts the intracellular localization of GluR1 in a similar way to Dyn2. In cells overexpressing or lacking Ndel1, the ER and TGN distribution remain unaffected (Fig. S1). Thus, it is unlikely that the Ndel1 siRNA-mediated redistribution of GluR1 is due to remodelling of these organelles [20]. However, we cannot exclude the possibility that Ndel1 affects the positioning and morphology of organelles other than the ER and TGN. It should also be noted that Dyn2 affects TGN biology as shown on Fig. S1C [31] but it is unknown whether this contributes to the redistribution of GluR1 in cells through a Ndel1-independent mechanism. As a third non-mutually exclusive scenario based on the interactions data, in vitro GTPase assays and comparative studies with Ndel1 siRNA and dominant negative Dyn2, we speculate that Ndel1, by regulating Dyn2 GTPase activity, could modulate the trafficking, i.e. vesicle-mediated transport, of GluR1. This speculative model finds support in the literature. Indeed, by virtue of its interactions with Dyn2 (this study), cytosolic proteins involved in membrane morphogenesis such as αCOP [4], cytoskeletal proteins and molecular motors [2], [3], [12], [49], [50], Ndel1 would be a candidate of choice to integrate cytoskeleton dynamics and membrane fission during membrane trafficking. On the one hand, by increasing Dyn2 GTPase activity, Ndel1 could help the cytosolic enzyme to constrict membranes and sever GluR1-containing vesicles from heavy membranes. On the other hand, Ndel1 and Dyn2 have been linked to actin [14]–[17], [34]–[36], [38], [39] and Ndel1 can modulate actin organization and dynamics to provide the longitudinal tension necessary to pull apart membranes during fission [25]. Such longitudinal force has already been suggested in yeast in the form of localized actin polymerization at the site of endocytosis at the plasma membrane [27], [51], [52]. Alternatively, by regulating Dynein and Kinesin activities through Lis1 [5], [7], [12], [49], [50], [53] and by activating the walking of soluble molecular motors on MTs, Ndel1 could generate a similar longitudinal force through MTs. Ndel1 could also facilitate the polymerization of MTs to extend this force. This idea of longitudinal force generated by MTs/Dyn2/Ndel1 would be compatible with a recent study reporting that Dyn2 also controls MTs stability [23]. Furthermore, a vesicles population with both Dynein and Kinesin I bound that may be capable of bi-directional motility along MTs has been described [49]. Further studies are required to test this appealing speculative model of dual (constrictive and longitudinal) forces involving Ndel1/Dyn2/MTs and active transport of Dyn2 in mammalian cells.

### Ndel1 and other cellular functions associated with Dynamins

We have focused our study on the full length Dyn2 due to its ubiquitous nature in context of GluR1 trafficking. As a centrosomal protein, Ndel1 may participate in centrosome cohesion via binding to Dyn2 and gamma-Tubulin [40]. The Dyn family contains 3 members (Dyn1, Dyn2, Dyn3) expressed in a tissue-specific manner [22]. The alternative splicing sites in the *Dynamin* gene further increase the diversity of Dyn proteins in cells. So far, more than 20 Dyn isoforms have been reported [54]. Therefore, Ndel1 may also interact with other Dyn members and isoforms to control cellular functions such as clathrin-mediated receptor endocytosis, calveolae internalization, vesicles recycling at the synapse and lamellipodia formation [33]–[39], [41].

Finally, Ndel1 has been extensively studied in regards to its essential role in brain development and maturation. Its association with the MT-associated factors Lis1 and DISC-1 (the lissencephaly and schizophrenia-causing proteins, respectively), has incriminated it as a potential candidate in developmental and neuropsychiatric disorders. The findings that Ndel1 acts on the functions of intermediate filament protein NF-L [1] and Dyn2 (this study), both mutated in the sensory motor neurodegenerative Charcot-Marie-Tooth disease [55]–[58] further highlight his key role in degenerating neurons. Thus, investigation of the roles of Ndel1/Dyn complex in nerve cells will provide a better understanding of the importance of cytoskeleton/membrane interface in the healthy and diseased nervous system.

## Materials and Methods

### Immunoprecipitations

Cells were lysed in lysis buffer (50 mM Tris–HCl at pH 7.4, 150 mM NaCl, 10 mM KCl, 1 mM EDTA, 0.5% NP40, 0.5% Tween-20 containing a protease inhibitor cocktail (Roche Diagnostics)) by sonication. Supernatants were incubated with appropriate antibodies and the resulting immune complexes were washed 4 times with lysis buffer, separated by SDS–PAGE and immunoblotted.

### Far-western and in vitro pull-down assays

Far-western assays were performed with affinity-purified GST-fused Dyn2 and His-tagged Ndel1 proteins. GST-fused Dyn2 (*M*_r_ of ∼130 kDa) and His-tagged Ndel1 (*M*_r_ of ∼45 kDa) proteins were expressed in BL-21 *Escherichia coli* (Stratagene) cells and purified on a Glutathione Sepharose 4 Fast Flow column (GE Healthcare) or Ni-NTA Agarose column (Qiagen) according to the manufacturer's protocol. The in vitro pull-down assays of the GST-fused Dyn2 and His-tagged Ndel1 were carried out with Glutathione Sepharose 4 Fast Flow beads or Ni-NTA Agarose beads in 500 µL of binding buffer containing 20 mM HEPES, 150 mM NaCl, 1 mM DTT, a protease inhibitor cocktail and 0.1% bovine serum albumin (BSA) at 4°C for 2 hours.

### Yeast two-hybrid assays

Dyn2 full length isoform was amplified by PCR and inserted into the *Sal*I and *Not*I sites of pPC86 vector (GAL4-activation-domain) (Invitrogen). Full-length (a.a. 1–345), coiled-coil domain (a.a. 1–201) and carboxyl tail (a.a. 191–345) of Ndel1 were cloned into pPC97 vector (GAL4-DNA-binding-domain). MaV203 yeast cells were co-transformed with various constructs of pPC97 and pPC86 vectors. Colonies grown on SD-*Leu*/*Trp* media were streaked onto a YPD (yeast peptone dextrose) plate and colony-lifting assays for β-galactosidase expression were carried out according to the manufacturer's instructions (Clontech). Also, transformants were plated on SD-*Leu*/*Trp*/His media, containing 20 mM or 40 mM 3-amino-1,2,4-triazol (3-AT) and incubated for 5 days at 30°C.

### Cell culture, transfection

HeLa cells (ATCC) were cultured at 37°C, 5% CO_2_ in DMEM supplemented with 10% fetal bovine serum. For the transfection and transient expression of proteins (Dyn2-GFP, Dyn2(K44A)-GFP, Flag-tagged Ndel1, GluR1 and ER-mCherry (see below)), cells were transfected with Lipofectamine 2000 (Invitrogen) according to the manufacturer's instructions. HeLa cells were transfected with synthetic siRNAs (25–100 nM, duplexes) specific for Ndel1 (5′- GCAGGUCUCAGUGUUAGAA -3′) by using HiPerfect transfection reagent (Qiagen) according to the manufacturer's instructions and then cultured for 24 hours or 48 hours to achieve Ndel1 silencing. Allstars negative control siRNA duplexes (Qiagen) were used as a control. The molar ratios of GluR1 construct to other DNA constructs or Ndel1 siRNAs for co-transfection were 1∶1 for biochemical analyses. For ER structure experiments, HeLa cells were co-transfected with ER-mCherry and control or Ndel1 siRNA using Lipofectamine 2000. The total number of cells analyzed for classifications of described ER phenotypes exceeded 200 for control siRNA-treated cells and 450 for Ndel1 siRNA-treated cells.

### Immunocytochemistry

Briefly, HeLa cells were fixed for 20 minutes in warm PBS with 4% paraformaldehyde and permeabilized with 0.5% saponin. They were incubated in 10% goat serum (2.5 hours, 25°C), appropriate primary antibody in PBS with 3% BSA, 0.5% saponin (overnight, 4°C) and secondary antibody in PBS with 3% BSA, 0.5% saponin (2 hours, 25°C). The immunofluorescent staining was performed with GluR1 (Chemicon International), Ndel1 (custom-made) and p230 trans-Golgi (BD Biosciences) antibodies. For ER staining, cells were transfected with an ER-targeted fluorophore, ER-mCherry, produced using variations of previously reported methods [59], [60]. PCR primers complementary to the mCherry sequence of the mCherry vector in pcDNA 3.1(+) (a generous gift from Dr Michael A Colicos) were used to amplify the mCherry sequence and add a *BamH*I restriction site on 5′ end and the KDEL ER retention signal plus *EcoR*I restriction site on 3′ end. An ER targeting peptide, corresponding to the 17 initial amino acids of Calreticulin was synthesized and *Nhe*I and *BamH*I restrictions sites were added on 5′ and 3′ end respectively. Both inserts were further ligated between the *Nhe*I and *EcoR*I restriction sites of the pcDNA 3.1(+) (Invitrogen) under control of the CMV promoter. Confocal images were captured with a Nikon Eclipse C1 laser and detector units mounted on a Nikon Eclipse TE2000-E microscope with a 60X 1.40 oil objective using EZ-C1 3.50 imaging software.

### Post-mitochondrial fractions preparation

Briefly, post-mitochondrial fractions (PMF) from HeLa cells were isolated using a series of centrifugations and homogenization/resuspension in hypotonic/isotonic extraction buffers (for additional details, see the manufacturer's protocol for isolation kit ER0100; Sigma-Aldrich). The PMF is the source of ER microsomes and is also enriched in plasma membrane, Golgi membranes and small vesicles/endosomes membranes (Fig. 1C). The PMF was examined by Western blot analysis using Tubulin (Sigma-Aldrich), p230 trans-Golgi, EEA1 (BD Biosciences), Na^+^/K^+^ATPase (Upstate), BiP and KDEL ER marker (Santa Cruz Biotechnologies) antibodies.

### Preparation of heavy and light membrane fractions by velocity sucrose gradient centrifugation

The preparation was performed according to reference [61]. Briefly, 2×10^7^ cultured HeLa cells transfected with or without Dyn2-GFP, Dyn2(K44A)-GFP, Flag-tagged Ndel1, Ndel1 siRNA and GluR1 constructs were washed twice with ice-cold PBS and re-suspended in 0.5 mL ice-cold Homogenization Buffer (0.2 M sucrose, 10 mM HEPES, 1 mM EDTA, protease inhibitor cocktail (Roche Diagnostics) pH 7.4). The nuclei and unbroken cells were pelleted by centrifugation at 1,000× *g* for 10 minutes after homogenization. The supernatant (∼1 mL) was loaded onto a 0.2 M to 1.2 M linear sucrose gradient and centrifuged at 25,000 r.p.m. in a Beckman rotor (SW41) for 15 minutes (after reaching the final speed). Five millilitres from the top of the gradient were collected as the light membrane fraction (LM). Four millilitres from the bottom of the gradient were collected as the heavy membrane fraction (HM). The collected fractions were subjected to buffer change by ice-cold Homogenization Buffer without sucrose and concentrated by an Amicon Ultra centrifugal filter device (Millipore). All steps were carried out at 4°C.

### Dynamin 2 GTPase assays

Radioactive GTPase assays were conducted in Assay buffer (10 mM HEPES, 10 mM PIPES, 2 mM MgCl_2_, 1 mM EGTA, 1 mM DTT at pH 7.0, 0.1% BSA) containing 0.5 mM GTP in a final volume of 20 µL. Bacterially-expressed Dyn2 (1.5 µM) was preincubated with or without purified His-Ndel1 protein for 30 minutes at room temperature. Reactions were initiated by adding GTP spiked with 0.5 µCi α-^32^P-GTP and incubated at 37°C. Aliquots (1.5 µL) were removed at specified times and spotted onto polyethyleneimine (PEI) cellulose thin-layer chromatography (TLC) plates (Scientific Absorbents Incorporated). Nucleotides were resolved by thin layer chromatography in 1∶1 ratio of 1 M LiCl: 2 M formic acid solution. The TLC plates were dried in a warm air drier and autoradiographed with HyBlot CL film (Denville Scientific Inc.) for 4 hours at −80°C. GTP and GDP levels were quantified using the Labscan program (Image Master, 2D software v3.10; Amersham Pharmacia Biotech). Percentages of GTP hydrolysis were calculated for a minimum of three time points, and were defined as (GDP/(GTP+GDP))×100.

Alternatively, the non-radioactive ELIPA GTPase assay (Cytoskeleton, Inc.) was used in accordance with the manufacturer's instructions. Bacterially-expressed Dyn2 constructs (0.8 µM) were preincubated with or without purified Ndel1 (1.5 µM) in Assay Buffer (10 mM HEPES, 10 mM PIPES, 2 mM MgCl_2_, 1 mM EGTA, 1 mM DTT at pH 7.0, 0.1% BSA) for 20 minutes at 37°C. Reactions were initiated by adding 1.5 mM GTP and incubated at 37°C. OD 360 nm was measured at regular intervals using a SpectraMax Plus^384^ (Molecular Devices) for 60 minutes. This assay evaluates GTPase activity by assessing the amount of inorganic phosphate (Pi) generated through GTP hydrolysis.

### Sedimentation assay

Dyn2 self-assembly with or without Ndel1 was tested by sedimentation after high-speed centrifugation. Dyn2 (2 µM) with or without Ndel1 (2 µM) was incubated in 10 mM HEPES, 10 mM PIPES, 2 mM MgCl_2_, 1 mM EGTA, 1 mM DTT at pH 7.0, 0.5 mM GTP in a final volume of 50 µL at 37°C for 15 minutes. Mixtures were then spun at 16,000 r.p.m. (20,800× g) for 20 minutes in a microfuge refrigerated at 4°C to obtain supernatant (S) and pellet (P) fractions. The pellet fraction was resuspended in 50 µL of the same buffer to obtain equal volumes of supernatant and pellet fractions. Samples were heated at 95°C for 5 minutes after addition of 10 µL of 6 X SDS–PAGE buffer, resolved on a 8% polyacrylamide gel and visualized by Coomassie staining.

## Supporting Information

Figure S1 Ndel1 depletion does not alter the distribution of the trans-Golgi network and endoplasmic reticulum. (A) Confocal pictures of HeLa cells transfected with a control siRNA or Ndel1 siRNA and stained for Ndel1 and p230 trans-Golgi, a protein associated with the trans-Golgi network (TGN). The depletion of Ndel1 does not alter the structure and intracellular localization of the TGN. Scale bar, 5 µm. (B) Confocal pictures of HeLa cells transfected with a control siRNA or Ndel1 siRNA and co-transfected with a construct encoding an endoplasmic reticulum (ER)-targeted fluorophore (ER-mCherry). The three upper panels represent the three ER phenotypes observed in both treatments: perinuclear, partially dispersed and fully dispersed ER. The bar graph shows the distribution of ER phenotypes among cells. Note that the distribution of the ER, labelled with the ER-mCherry remains largely unchanged in Ndel1 siRNA-transfected cells when compared to control siRNA-transfected cells. The bar graph reports the results of one experiment and is representative of the data found in 3 independent experiments. Chi-square analysis. ns, not significant. Scale bar, 10 µm. (C) Analysis by Western blots of the content of the KDEL ER marker and TGN marker p230 trans-Golgi in the light and heavy membrane fractions (LM and HM respectively) isolated from cells overexpressing GluR1 together with either Dyn2, a mutant inactive form of Dyn2 (Dyn2(K44A)), Ndel1, or depleted of Ndel1 by siRNA. Note that Ndel1 does not affect the distribution of the KDEL ER marker and TGN marker among the fractions: p230 is for instance mostly found in the HM fraction of cells overexpressing or lacking Ndel1 in a similar way to untransfected control cells. Dyn2, which is important for TGN biology affects p230 trans-Golgi fractionation [31] but not KDEL ER marker distribution. On the contrary, the inactive Dyn2(K44A) mutant does not affect p230 trans-Golgi distribution.
(7.83 MB TIF)
