# Supplementary figures and images for: The Cytoskeletal Protein Ndel1 Regulates Dynamin 2 GTPase Activity
Source: PLoS One. 2011 Jan 25;6(1):e14583. doi: 10.1371/journal.pone.0014583 (PMC3026782; doi:10.1371/journal.pone.0014583)

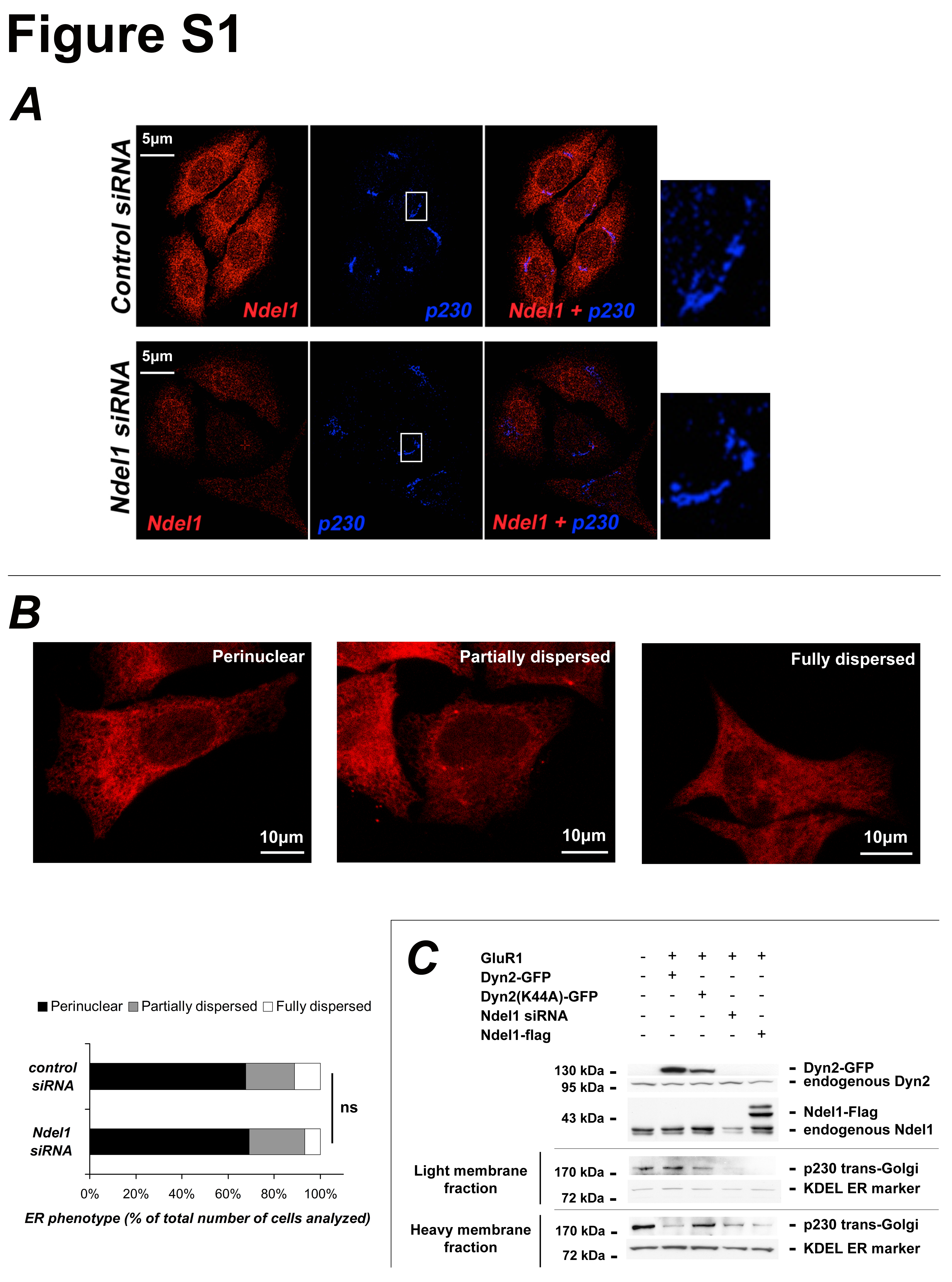

Supplement: Figure S1 — Ndel1 depletion does not alter the distribution of the trans-Golgi network and endoplasmic reticulum. (A) Confocal pictures of HeLa cells transfected with a control siRNA or Ndel1 siRNA and stained for Ndel1 and p230 trans-Golgi, a protein associated with the trans-Golgi network (TGN). The depletion of Ndel1 does not alter the structure and intracellular localization of the TGN. Scale bar, 5 µm. (B) Confocal pictures of HeLa cells transfected with a control siRNA or Ndel1 siRNA and co-transfected with a construct encoding an endoplasmic reticulum (ER)-targeted fluorophore (ER-mCherry). The three upper panels represent the three ER phenotypes observed in both treatments: perinuclear, partially dispersed and fully dispersed ER. The bar graph shows the distribution of ER phenotypes among cells. Note that the distribution of the ER, labelled with the ER-mCherry remains largely unchanged in Ndel1 siRNA-transfected cells when compared to control siRNA-transfected cells. The bar graph reports the results of one experiment and is representative of the data found in 3 independent experiments. Chi-square analysis. ns, not significant. Scale bar, 10 µm. (C) Analysis by Western blots of the content of the KDEL ER marker and TGN marker p230 trans-Golgi in the light and heavy membrane fractions (LM and HM respectively) isolated from cells overexpressing GluR1 together with either Dyn2, a mutant inactive form of Dyn2 (Dyn2(K44A)), Ndel1, or depleted of Ndel1 by siRNA. Note that Ndel1 does not affect the distribution of the KDEL ER marker and TGN marker among the fractions: p230 is for instance mostly found in the HM fraction of cells overexpressing or lacking Ndel1 in a similar way to untransfected control cells. Dyn2, which is important for TGN biology affects p230 trans-Golgi fractionation [31] but not KDEL ER marker distribution. On the contrary, the inactive Dyn2(K44A) mutant does not affect p230 trans-Golgi distribution. (7.83 MB TIF) [file pone.0014583.s001.tif]
